# Supplementary material for: cGAS-like receptors sense RNA and control 3′2′-cGAMP signalling in Drosophila
Source: Nature. Author manuscript; Available in PMC 2021 Sep 4. (PMC8410604; doi:10.1038/s41586-021-03743-5)
Supplement: Slavik et al SI Table 1 [file NIHMS1732820-supplement-Slavik_et_al_SI_Table_1.pdf]

Supplementary Table 1. Summary of data collection, phasing and refinement statistics

	hMB21D2 (7LT1)	hMB21D2 SeMet	*Tc*-cGLR (7LT2)	*Tc*-cGLR SeMet
**Data collection**				
Space group	I 2 2 2	I 2 2 2	P 2_1 2_1 2_1	P 2_1 2_1 2_1
Cell dimensions				
a, *b*, *c* (Å)	102.17, 127.82, 198.24	102.15, 127.20, 197.60	57.40, 70.44, 104.60	56.58, 70.67, 103.49
α, β, γ (°)	90.0, 90.0, 90.0	90.0, 90.0, 90.0	90.00, 90.00, 90.00	90.00, 90.00, 90.00
Resolution (Å)	49.56–2.40 (2.48–2.40)	49.40–2.99 (3.17–2.99)	41.99–1.58 (1.61–1.58)	44.17–1.65 (1.68–1.65)
R_{pim}	3.8 (82.3)	4.9 (45.8)	3.7 (61.2)	2.1 (81.6)
$I / \sigma(I)$	12.6 (1.3)	17.3 (2.8)	9.6 (1.1)	23.8 (1.3)
Completeness (%)	99.6 (99.8)	99.5 (97.3)	98.4 (98.1)	100.0 (100.0)
Redundancy	7.0 (6.9)	53.0 (49.9)	2.7 (2.6)	53.5 (43.3)
**Refinement**				
Resolution (Å)	49.56–2.40	–	41.99–1.58	–
No. reflections				
Total	353973	1398790	152854	2712556
Unique	50814	26374	57602	50722
Free	1983	–	2000	–
R_{work} / R_{free}	24.8 / 26.9	–	18.4 / 21.4	–
No. atoms				
Protein	6398	–	3187	–
Ligand/ion	15 (SO_4)	–	1 (Mn^{2+})	–
Water	–	–	543	–
B-factors				
Protein	97.0	–	25.6	–
Ligand/ion	130.4	–	34.0	–
Water	–	–	36.5	–
R.m.s. deviations				
Bond lengths (Å)	0.002	–	0.004	–
Bond angles (°)	0.40	–	0.68	–

*All datasets were collected from individual crystals. *Values in parentheses are for the highest resolution shell.

Supplementary Table 1. Summary of data collection, phasing and refinement statistics

	dSTING<br><br>**(7MWY)**	dSTING<br>SeMet	dSTING–<br>3′2′-cGAMP<br>**(7MWZ)**	dSTING–<br>3′2′-cGAMP<br>SeMet
**Data collection**				
Space group	P 3_2 2 1	P 3_2 2 1	P 1 2_1 1	P 1 2_1 1
Cell dimensions				
a, *b*, *c* (Å)	135.28, 135.28, 60.06	135.68, 135.68, 59.93	50.92, 95.05, 140.14	51.05, 95.71, 142.31
α, β, γ (°)	90.0, 90.0, 120.0	90.0, 90.0, 120.0	90.00, 93.93, 90.00	90.00, 94.57, 90.00
Resolution (Å)	44.91–1.84 (1.88–1.84)	44.92–2.40 (2.49–2.40)	48.84–2.00 (2.04–2.00)	49.16–2.89 (3.05–2.89)
R_{pim}	3.5 (65.6)	3.7 (36.2)	3.2 (75.9)	4.4 (35.9)
$I / \sigma(I)$	11.2 (1.1)	19.4 (3.1)	10.7 (1.2)	11.4 (2.9)
Completeness (%)	99.7 (98.6)	99.7 (98.5)	99.7 (99.1)	99.7 (98.3)
Redundancy	5.8 (5.8)	31.4 (31.1)	7.1 (7.1)	28.1 (26.4)
**Refinement**				
Resolution (Å)	44.91–1.84	–	48.84–2.00	–
No. reflections				
Total	318531	785751	639566	857564
Unique	54801	25033	89677	30535
Free	2005	–	2006	–
R_{work} / R_{free}	18.0 / 19.4	–	24.7 / 28.3	–
No. atoms				
Protein	2845	–	10943	–
Ligand/ion	–	–	90 (3′2′-cGAMP)	–
Water	399	–	511	–
B-factors				
Protein	39.23	–	62.6	–
Ligand/ion	–	–	45.0	–
Water	48.12	–	53.2	–
R.m.s. deviations				
Bond lengths (Å)	0.014	–	0.002	–
Bond angles (°)	1.23	–	0.44	–

*All datasets were collected from individual crystals. *Values in parentheses are for the highest resolution shell.
