# Supplementary material for: cGAS-like receptors sense RNA and control 3′2′-cGAMP signalling in Drosophila
Source: Nature. 2021 Jul 14;597(7874):109–13. doi: 10.1038/s41586-021-03743-5 (PMC8410604; doi:10.1038/s41586-021-03743-5)
Supplement: Supplementary file 1 [file 41586_2021_3743_MOESM1_ESM.pdf]

nature portfolio

Corresponding author(s): Philip Kranzusch

Last updated by author(s): Jun 11, 2021

Reporting Summary

Nature Portfolio wishes to improve the reproducibility of the work that we publish. This form provides structure for consistency and transparency in reporting. For further information on Nature Portfolio policies, see our Editorial Policies and the Editorial Policy Checklist.

Statistics

For all statistical analyses, confirm that the following items are present in the figure legend, table legend, main text, or Methods section.

n/a	Confirmed	
☐	☒	The exact sample size (*n*) for each experimental group/condition, given as a discrete number and unit of measurement
☒	☐	A statement on whether measurements were taken from distinct samples or whether the same sample was measured repeatedly
☐	☒	The statistical test(s) used AND whether they are one- or two-sided<br>*Only common tests should be described solely by name; describe more complex techniques in the Methods section.*
☒	☐	A description of all covariates tested
☒	☐	A description of any assumptions or corrections, such as tests of normality and adjustment for multiple comparisons
☐	☒	A full description of the statistical parameters including central tendency (e.g. means) or other basic estimates (e.g. regression coefficient) AND variation (e.g. standard deviation) or associated estimates of uncertainty (e.g. confidence intervals)
☐	☒	For null hypothesis testing, the test statistic (e.g. *F*, *t*, *r*) with confidence intervals, effect sizes, degrees of freedom and *P* value noted<br>*Give P values as exact values whenever suitable.*
☒	☐	For Bayesian analysis, information on the choice of priors and Markov chain Monte Carlo settings
☒	☐	For hierarchical and complex designs, identification of the appropriate level for tests and full reporting of outcomes
☒	☐	Estimates of effect sizes (e.g. Cohen's *d*, Pearson's *r*), indicating how they were calculated

Our web collection on statistics for biologists contains articles on many of the points above.

Software and code

Policy information about availability of computer code

Data collection	All radioactivity-based imaging was collected using Typhoon scanner control 2.0.0.6<br>Chromatography traces collected using GE Unicorn 7.1<br>Protein, DNA, and RNA gel images collected using BioRad ImageLab 2.4.0.3<br>Protein homologs identified using NCBI PSI-BLAST (web-based: https://blast.ncbi.nlm.nih.gov/Blast.cgi)
Data analysis	Phenix 1.19, Coot 0.8.9, PyMOL 2.3, GraphPad Prism 9.0.1, Geneious Prime v2020.12.23, Image Quant 8.2.0

For manuscripts utilizing custom algorithms or software that are central to the research but not yet described in published literature, software must be made available to editors and reviewers. We strongly encourage code deposition in a community repository (e.g. GitHub). See the Nature Portfolio guidelines for submitting code & software for further information.

Data

Policy information about availability of data

All manuscripts must include a data availability statement. This statement should provide the following information, where applicable:

- Accession codes, unique identifiers, or web links for publicly available datasets
- A description of any restrictions on data availability
- For clinical datasets or third party data, please ensure that the statement adheres to our policy

Atomic coordinates and structure factors of human MB21D2, T. castaneum cGLR, Drosophila STING, and the Drosophila STING–3'2'-cGAMP complex have been deposited in PDB under the accession codes 7LT1, 7LT2, 7MWY, and 7MWZ. All other data are available in the manuscript or the supplementary materials.

Field-specific reporting

Please select the one below that is the best fit for your research. If you are not sure, read the appropriate sections before making your selection.

☒ Life sciences ☐ Behavioural & social sciences ☐ Ecological, evolutionary & environmental sciences

For a reference copy of the document with all sections, see nature.com/documents/nr-reporting-summary-flat.pdf

Life sciences study design

All studies must disclose on these points even when the disclosure is negative.

Sample size	Sample size for all Drosophila experiments was determined using previously published protocols (Cai et al., 2020, PMID:33262294).
Data exclusions	No data were excluded from analyses
Replication	All experiments were performed with independent replicates as described in the figure legends.
Randomization	X-ray crystal structures were refined with a randomly selected R-free reflection set based on automatic selection in Phenix 1.19. Flies were randomly selected for injection with any of the tested CDNs or buffer control. No other randomization was required for the cell biological, biochemical, and structural analyses in this study.
Blinding	Blinding was not performed for data analysis or group allocation for Drosophila experiments. Flies were randomly selected for each experimental group and data were collected by unbiased, quantitative means.

Reporting for specific materials, systems and methods

We require information from authors about some types of materials, experimental systems and methods used in many studies. Here, indicate whether each material, system or method listed is relevant to your study. If you are not sure if a list item applies to your research, read the appropriate section before selecting a response.

Materials & experimental systems

n/a	Involved in the study
☒	☐ Antibodies
☐	☒ Eukaryotic cell lines
☒	☐ Palaeontology and archaeology
☐	☒ Animals and other organisms
☒	☐ Human research participants
☒	☐ Clinical data
☒	☐ Dual use research of concern

Methods

n/a	Involved in the study
☒	☐ ChIP-seq
☒	☐ Flow cytometry
☒	☐ MRI-based neuroimaging

Eukaryotic cell lines

Policy information about cell lines

Cell line source(s)	HEK 293T (catalog ATCC CRL-3216) cells were purchased directly from ATCC.
Authentication	HEK 293T cells were validated by ATCC
Mycoplasma contamination	Cell lines were not tested for mycoplasma contamination.
Commonly misidentified lines (See ICLAC register)	No misidentified lines were used.

Animals and other organisms

Policy information about studies involving animals; ARRIVE guidelines recommended for reporting animal research

Laboratory animals	All Drosophila melanogaster fly lines are described in methods, were handled according to standards practices in the field, and are Wolbachia free. Equal numbers of male and female flies were selected for each experimental group. Flies were 3-5 days old at the commencement of each experiment.
Wild animals	No wild animals were used in this study.

Field-collected samples	No field-collected samples were used in this study.
Ethics oversight	No ethics oversight was required for this study.

Note that full information on the approval of the study protocol must also be provided in the manuscript.
